# Health-related quality of life in Hong Kong physicians up to 20 years post-graduation: A cross-sectional survey

**DOI:** 10.1371/journal.pone.0284253

**Published:** 2023-04-12

**Authors:** Amy Pui Pui Ng, Weng Yee Chin, Eric Yuk Fai Wan, Julie Chen, Chak Sing Lau

**Affiliations:** 1 Department of Family Medicine and Primary Care, The University of Hong Kong-Shenzhen Hospital, Futian District, Shenzhen, Guangdong Province, China; 2 Department of Family Medicine and Primary Care, Li Ka Shing Faculty of Medicine, The University of Hong Kong, Ap Lei Chau, Hong Kong; 3 Bau Institute of Medical and Health Sciences Education, Li Ka Shing Faculty of Medicine, The University of Hong Kong, Hong Kong, Hong Kong; 4 Department of Medicine Hong Kong, Li Ka Shing Faculty of Medicine, The University of Hong Kong, Hong Kong, Hong Kong; 5 Department of Pharmacology and Pharmacy, LKS Faculty of Medicine, HKU, Pokfulam, Hong Kong SAR; Jouf University, UNITED KINGDOM

## Abstract

**Background:**

Problems with health-related quality of life can affect physicians’ ability to work effectively. This study compared the health-related quality of life of Hong Kong physicians to the general population and explored the factors associated with mental and physical health-related quality of life.

**Methods:**

This cross-sectional study was conducted from January to April 2016. Medical graduates from the University of Hong Kong participated in a survey containing the Short Form-12 Item Health survey version 2, Patient Health Questionnaire-9, Copenhagen Burnout Inventory, and items on lifestyle behaviors, career satisfaction, and socio-demographics.

**Results:**

496 responses were received. The mean physical component summary score was 53.2 (SD = 7.6), similar to the general population. The mean mental component summary score was 43.6 (SD = 11.8), significantly worse than the general population (P<0.01). Compared to the general population, all Short-Form 12 Health Survey version 2 domains were worse in doctors, aside from bodily pain and general health. Regular exercise was positively associated with physical component summary scores (Coeff 2.024; P = 0.047); but having children and higher personal burnout scores were negatively associated with it (Coeff -1.890; P = 0.036; and Coeff -0.045; P = 0.027, respectively). Poorer mental component summary scores correlated with worse personal (Coeff -0.284; P< 0.001), work-related (Coeff -0.135; P = 0.040), and patient-related burnout (Coeff -0.060; P = 0.041), and higher Patient Health Questionnaire-9 scores (Coeff -9.170; P<0.001). There were significant differences in mental health (P = 0.042) and mental component summary scores (P = 0.012) across age groups, but not with gender.

**Conclusion:**

Hong Kong physicians are less impacted by physical health than mental health. Compared to the general population, doctors’ mental health has a more significant impact on their lives. Interventions aimed to improve burnout and depression rates in physicians may improve physicians’ mental health-related quality of life.

## Introduction

It is widely reported that being a physician is stressful [1]. Studies have shown that physicians experience higher rates of mental health problems than the general population [2, 3]. A meta-analysis of 54 studies estimated the prevalence of depression or depressive symptoms among resident physicians to be 28.8% [4], much higher than the WHO global estimate of 2.6% to 5.9% [5]. International studies have shown that the prevalence of burnout in physicians ranges from 8% to 68.2% [6–8]. Poor clinician mental health can lead to poorer patient outcomes due to reduced work effort [9], increased absenteeism [2, 10], higher intention to leave work [11], more medical errors [2, 12], and increased suicide risk [2].

Physicians are more likely to self-report that they are physically healthier than the general population [13–16]. However, global research from both developed and developing countries on the prevalence of chronic illnesses in physicians compared to the general population has been mixed, with some reporting a lower [17, 18] and others reporting a higher [19, 20] prevalence of chronic diseases in physicians. Physicians also tend to have lower mortality and live longer than the general population [21]. Concerning health behaviors, some studies have found that doctors often fail to follow preventative health guidelines and are more likely to drink alcohol [22, 23]. However, others have found that physicians smoke less, exercise more, have better diets, and are less likely to binge drink [17, 22]. Doctors with chronic diseases have poorer outcomes. Chronic illnesses and physical health problems are associated with increased sick leave and limitations at work in the short and long term [13, 24] and increased risk of all-cause mortality [25].

Focusing on physician health is essential as doctors are critical players in the medical system and having good health is essential for the performance of healthcare workers [26]. Health-related quality of life (HRQoL) is a commonly used outcome measure that enables us to understand the impact of illness. It examines how changes in health affect people’s daily functioning and if they restrict daily activities [27], such as their work. It assesses perceived well-being in the physical, mental, and social domains of health [28], measures the gains from health interventions or policy changes and helps policymakers make decisions regarding resource allocation. Physicians’ HRQoL may directly or indirectly affect healthcare quality and safety, making it worth studying. The findings from physician HRQoL studies have been variable, with some indicating that physicians have only poor mental HRQoL [29–31], while others report that physicians suffer from only poor physical HRQoL [27]. Adding to the controversy, some studies have found physicians have poor mental and physical HRQoL [32]. In addition, physician studies have shown that mental HRQoL is associated with burnout, gender, and marital status [33, 34]. Other factors associated with HRQoL in physicians have included career satisfaction [35], number of shifts [32], religion [36], social relations [36], depression [37], stress [37], sleep quality [38], physical exercise [38], and satisfaction with family [38].

Although there have been several studies assessing HRQoL in medical professionals, there have been inconsistencies with the instruments used. 36-Item Short Form Survey (SF-36) [27, 30, 34, 38] and The World Health Organization Quality of Life: Brief Version (WHOQOL-BREF) [32, 35, 39] are commonly used in physician studies, but they appear to measure different constructs. The SF-36 measures HRQoL, while the WHOQOL-BREF measures quality of life more broadly [40]. We used the Short Form-12 item Health Survey (SF-12) for this study. The SF-12 is a shorter survey derived from the Medical Outcomes Study (MOS) 36-Item Short-Form Health Survey version 2 (SF-36v2). SF-12v2 has been used in other physician surveys [33], making comparisons to international studies possible. The Chinese version has been validated in Hong Kong (HK) with population norms available, making comparisons with the general HK population possible [41].

Research shows that doctors working in Asian countries have a higher prevalence of burnout than those in Western countries [6–8]. Our previous studies found 16% of HK physicians are depressed, 15.3% have thoughts of suicide or self-harm, and the prevalence of the specific subscales of burnout ranged from 35–63% [42, 43], indicating that there are significant mental health problems among physicians in HK. In addition, Chinese studies show a lower prevalence of some chronic illnesses but a higher prevalence of other chronic diseases than the general population [44]. Health habits are also different in China, such as having a higher prevalence of smoking and at-risk alcohol consumption among Mainland Chinese physicians [45, 46] than among physicians in HK [42, 43]. HRQoL research of physicians in Mainland China and HK supports relatively better physical HRQoL but poorer mental HRQoL [29–31]. There has been only one study to date reporting HRQoL in HK physicians. It found that both physical and mental HRQoL in young HK physicians was poorer than in the general population, and 32% of the physicians reported their self-perceived health was worse than their peers [31]. However, it did not look into the details of the specific domains of HRQoL and the factors associated with HRQoL. The factors affecting HK physicians’ HRQoL would likely differ from other countries. This is because HK’s environment is unique as it is greatly influenced by both Chinese and Western cultures. In addition, our previous research on burnout and depression in HK physicians showed different ways doctors manage their mental health problems, which may affect their HRQoL [42, 43]. Local data on doctors’ mental and physical HRQoL would provide insight into an inadequately explored subject that has ramifications for patient care and physician well-being.

### Aims

This study aimed to explore the HRQoL of HK physicians.

### Specific objectives

- To compare the HRQoL of life of HK physicians to the general population
- To explore the factors associated with mental and physical HRQoL in HK physicians.

## Methods

### Type of study

This is a cross-sectional study.

### Study setting

Between January 29 and April 15, 2016, subjects were contacted by email to complete an online survey in English. Following the initial invitation, two reminder emails were sent 14 days apart. Paper questionnaires were then delivered to graduates with available postal addresses to boost the sample size. As an incentive, respondents were given a coffee coupon.

### Study participants

**Fig 1:**
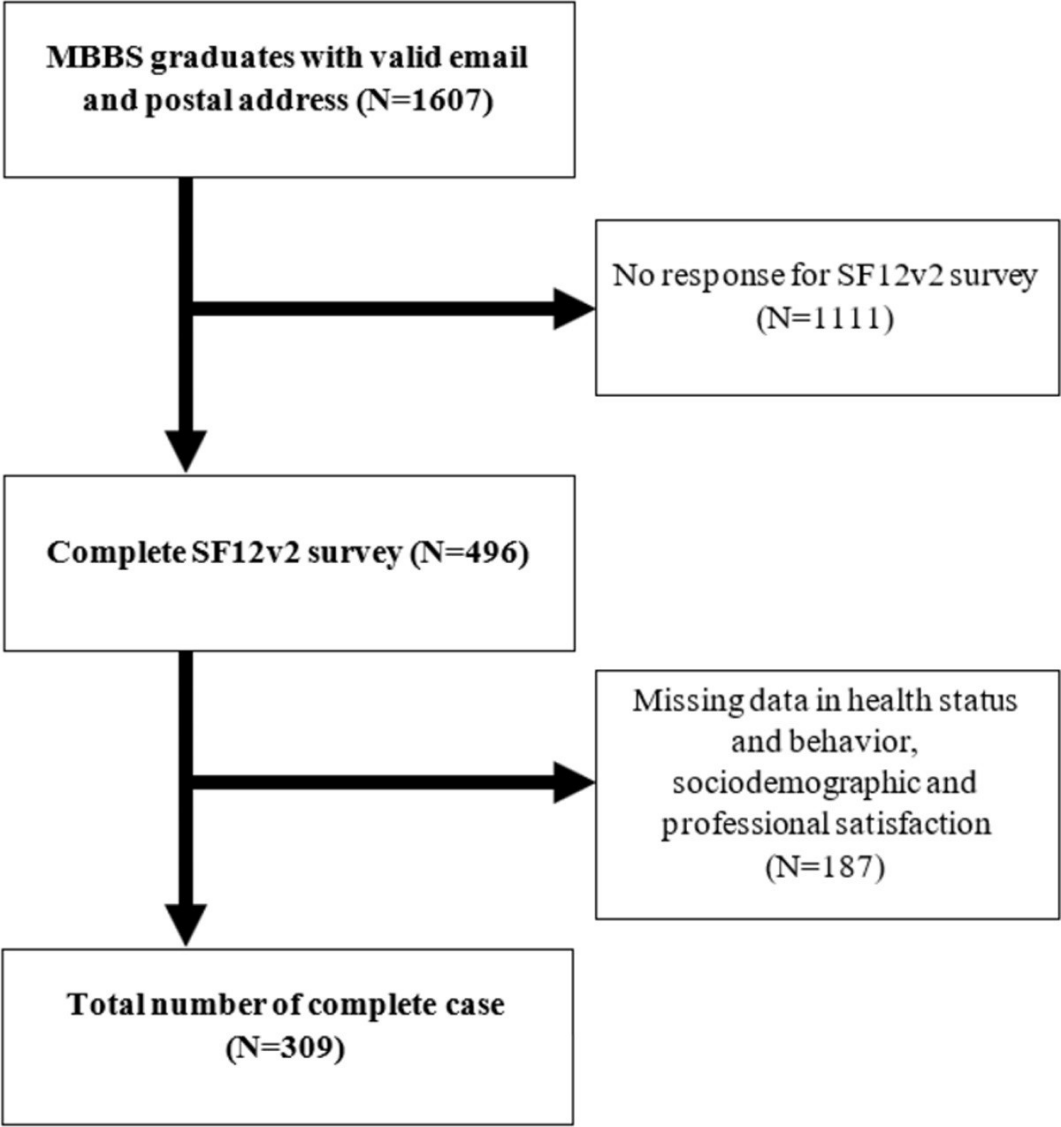
Flow chart showing the sampling and response rates. MBBS, Bachelor of Medicine, and Bachelor of Surgery. SF12v2, The Short Form 12-Item Health Survey version 2.

Medical school graduates from 1995 and 2014 from the University of Hong Kong with a valid email or mailing address recorded with the Faculty’s Alumni Office (N = 1607) were invited to participate in this cross-sectional survey by convenience sampling. The survey took 10–15 minutes to complete. Graduates without any mailing or email addresses were excluded. Among 1,607 eligible MBBS graduates, 496 completed the SF-12v2 survey (response rate of 30.8%). A total of 309 graduates with complete data were included (19.2%). The subject flow chart is shown in **Fig 1.**

### Study instruments

#### The short form 12-item health survey version 2

(SF-12 v2) was used to measure HRQoL in English. The SF-12 health survey is an abbreviated version of the SF-36 health survey, a popular and validated HRQoL measure [47]. The SF-12v2 has eight domains of HRQoL and two summary scales (a physical component (PCS) and a mental component (MCS)) [41]. The eight domains have questions related to (1) limitations in physical activities because of health problems (physical functioning (PF)); (2) limitations in usual role activities because of physical health problems (role physical (RP)); (3) bodily pain (BP); (4) general health perceptions (general health (GH)); (5) vitality (VT); (6) limitations in social activities because of physical or emotional problems (social functioning (SF)); (7) limitations in usual role activities because of emotional problems (role emotional (RE)); and (8) general emotional health (mental health (MH)). Each scale is scored from 0–100, with higher scores indicating better health. For the mental component summary (MCS) and physical component summary (PCS), the mean score is set at 50 with a standard deviation of 10. Scores > 50 indicate better physical or mental health than the mean [41]. This instrument has been used among overseas doctors [48]. A study in HK has shown that SF-12v2 is valid, reliable, and an equivalent substitute for SF-36.v2 with Cronbach alphas of 0.67 and 0.60 for the PCS and MCS scores, respectively [47].

#### The Copenhagen burnout inventory

(CBI) was used to measure burnout. The CBI is a 19-item questionnaire that measures exhaustion in three categories: personal, work-related, and patient-related. These items are evaluated on a five-point Likert scale: always or to a very high degree; often or to a high degree; sometimes or somewhat; seldom or to a low degree; and never/almost never or to a very low degree [10] with every response allocated a pre-determined number of points: 0, 25, 50, 75 and 100 respectively. The burnout level is computed as a mean score; therefore, every scale is scored between 0–100. Scores ≥50 indicate a high degree of burnout [6, 7, 49]. Cronbach’s alpha coefficients were measured in our previous study showing 0.92, 0.85, and 0.90 for personal, work-related, and patient-related subscales, respectively, providing evidence that the CBI was a reliable measure with high subscale internal consistency [43].

#### Patient health questionnaire-9

(PHQ-9) was used to calculate the prevalence of depression and severity of depression. It consists of nine items based on the DSM-V definition of depression. Each item is scored from zero (not at all) to three (nearly every day), with scores ranging from zero (no depressive symptoms) to 21 (severe depressive symptoms) [50]. In this study, a PHQ > 9 was used as the cut-off indicating the presence of depression, which has a sensitivity of 80% and specificity of 92% for the diagnosis of depression in HK [51]. PHQ-9 is a reliable and validated survey for depression and has a Cronbach’s alpha coefficient of 0.89 [52].

#### The alcohol use disorders identification test version C

(AUDIT-C) is a screening tool that assesses drinking behaviour using three questions. HK guidelines use a score > 3 as a positive screen for at-risk drinking [53]. The scores in this survey were taken from the original questionnaire and modified to the AUDIT-C scoring system. Cronbach’s alpha coefficient for AUDIT-C has previously been reported to be between 0.80–0.91 [54, 55].

#### Job satisfaction, health status, and behaviours

Survey questions on job satisfaction, health status, and lifestyle behaviours were adapted from existing doctor questionnaires [56] and HK population health surveys [57]. Variables were selected based on previous literature on factors affecting HRQoL, physical health, or mental health [6, 32–38, 48, 49, 58].

### Sample size

The sample size calculation was based on the rule of thumb that a minimum of 10 subjects for one potential risk factor is required for regression analysis [59]. Hence, 160 participants were needed to examine the 16 predictors in this study.

### Data management

To extract the data from the questionnaires, the responses were coded using number codes to make the data suitable for analysis. SoGo Survey was used for data entry. Only one database was created. The database had one variable for each item in the questionnaire and one record for each. Each participant had a unique identification number. The file was then exported to excel format, and then the dataset was converted to Stata format for statistical analysis. One of the authors (EW) and his trained research assistants handled the data to ensure consistency in the coding and analysis of the data. Only one research assistant helped with the statistical analysis of this paper, and EW checked the accuracy of the analyses. To address missing data, surveys with incomplete SF-12v2 items were removed from the analysis. Then, survey responses that had missing data on health status and behavior, sociodemographic and professional satisfaction were also removed.

### Data analysis

Descriptive statistics were used to summarize the respondents’ health status and behavior, sociodemographic characteristics, and professional satisfaction. Variance and t-tests were used to analyze the effect of age and gender on HRQoL. When more than two groups were compared, we performed post hoc pairwise comparisons with a reference group using the Scheffe method. Independent group t-tests were conducted to compare the doctors’ mean scores of the SF-12v2 domains, PCS, and MCS with the age-sex-adjusted HK population, which was collected from the previous population norm of SF12-v2 in Hong Kong [60]. The effect size was calculated using Cohen’s d formula with pooled standard deviation for the independent t-test. Effect sizes of 0.2 indicated a small, 0.5 a moderate, and 0.8 a large effect. Multivariable linear regression with backward selection was conducted to evaluate the associations between the factors (socio-demographic characteristics, professional satisfaction, and health status) and SF-12 scores.

All significance tests were conducted using the two-tailed 95% confidence interval, and findings with p-value <0.05 were considered significant. All analyses were performed using Stata Version 15.0 (StataCorp LP, College Station, TX, USA).

### Ethical considerations

The study was voluntary, and survey responses were taken as implied consent by agreeing to participate. The Institutional Review Board of the University of Hong Kong/ Hospital Authority Hong Kong West Cluster (UW 15–405) approved the study. The institutional review board approved implied consent because the risk of harm from the survey study was low, the population was not considered vulnerable, and the data collection was de-identified to remain anonymous. All research procedures were performed following regulations.

## Results

### Health status and behaviours, socio-demographics, and professional satisfaction characteristics

Subject characteristics, including health status and behaviours, socio-demographics, and professional satisfaction, are shown in **Table 1**. The mean age of respondents was 33.0 (SD = 5.5) years and 43.7% were female. Most respondents indicated they were satisfied with their current job position (77.7%) and their career choice as a doctor (94.8%). There were 24 participants in non-medical posts.

**Table 1:** Descriptive statistics on sociodemographic, professional satisfaction, and health status.

	Doctors (N = 309)		Doctors (N = 309)
**Health status and behaviour**		**Sociodemographic**	
At-risk drinker	47 (15.2%)	Age	33.0 ± 5.5
Current Smoker	2 (0.6%)	Gender (Female)	135 (43.7%)
Regular exercise	240 (77.7%)	Marital status	
Average sleep per night	6.7 ± 1.0	Single, separated and divorced	161 (52.1%)
Hours of work per week	55.2 ± 15.9	Married	148 (47.9%)
Depression	47 (15.2%)	Having children	103 (33.3%)
Burnout		Setting of practice	
Personal	58.1 ± 21.2	Public	258 (83.5%)
Work-related	49.2 ± 7.1	Private	51 (16.5%)
Patient-related	43.0 ± 21.4	Current specialty	
Number of chronic illnesses	0.2 ± 0.5	Orthopedic Surgery/ Otorhinolaryngology/ Surgery/ Ophthalmology/ Obstetrics & Gynaecology	98 (31.7%)
**Professional satisfaction**		Family Medicine/ General Practice/ Community Medicine, Emergency Medicine, Pediatrics	97 (31.4%)
Satisfied with present job position	293 (94.8%)	Intensive care, clinical oncology, dermatology and venereology, internal medicine, psychiatry	86 (27.8%)
Satisfied with being a medical doctor	240 (77.7%)	Pathology, radiology	28 (9.1%)

Notes:

Current Smoker (Current smoker vs Non-smoker/ex-smoker)

At-risk drinkers were defined if the doctors had 3 or more AUDIT-C scores. This AUDIT-C score was calculated by the sum of scores based on the frequency of drinking alcohol, the average number of standard drinks per day, and the frequency of taking 5 or more drinks on one occasion, ranging from 0 to 12. The frequency of drinking alcohol (Less than once per month/ Once per month/ 2 to 3 times per month or once per week/ 2 to 3 times per week/ 4 to 6 times per week or every day) ranks the score from 0 to 4 respectively; the average number of standard drinks per day (1–2 drinks/ 3–4 drinks/ 5–6 drinks/ 7–8 drinks/ 9 or more drinks) ranks the score from 0 to 4 respectively; the frequency of taking 5 or more drinks on one occasion (Never/ Less than once per week/ Monthly/ Weekly/ Daily or almost daily) ranks the score from 0 to 4 respectively.

Regular exercise (5 or more days per week for at least 10 minutes per day / Any vigorous and moderate physical activities)

Private Practice (Private Solo/ Private Hospital/Non-government organization)

Public Practice (University/Government/Hospital Authority/Not applicable)

All data are represented in mean ± SD or total (%), as appropriate.

No data is shown for missing values.

The prevalence of depression was 15.2%, and the mean PHQ-9 score was 5.3 (SD = 5.7). 63.4% had personal, 57.9% had work-related, and 35.9% had patient-related burnouts, with mean CBI scores of 58.1 (SD = 21.2), 49.2 (SD = 7.1), and 43.0 (SD = 21.4), respectively. 15.2% were at-risk drinkers. The mean number of chronic illnesses was 0.2 (SD = 0.5), and 77.7% of participants exercise regularly.

### SF-12v2 comparison with age-sex adjusted HK general population

**Table 2** compares doctors’ SF-12v2 domains, PCS, and MCS scores versus age-sex adjusted HK general population. Doctors’ mean MCS and PCS were 43.6 (SD = 11.8) and 53.2 (SD = 7.6), respectively. The highest-scoring domains of our participants were PF (91.0 (SD = 19.0)), followed by BP (84.5 (SD = 20.6)) and RP (76.5 (SD = 26.1)) and the lowest-scoring domains were VT (47.8 (SD = 22.4)) and GH (59.6 (SD = 25.0)). There was no significant difference in PCS between doctors’ (53.2 (SD = 7.6)) and the general population (52.5 (SD = 6.9)) (P = 0.142; effect size 0.10). However, MCS was significantly worse in doctors (43.6 (SD = 11.8) doctor group vs 48.6 (SD = 0.2) general population; P <0.001; effect size 0.48).

**Table 2:** Comparison of SF-12v2 domains, PCS, and MCS between doctors and age-sex-adjusted HK general population.

	Doctors	Age-sex-adjusted HK general population	P-value	Effect size^a^
Physical Functioning	91.0 ± 19.0	93.8 ± 15.2	0.014*	0.17
Role Physical	76.5 ± 26.1	82.8 ± 19.0	<0.001*	0.27
Bodily Pain	84.5 ± 20.6	79.9 ± 23.3	0.002*	0.21
General Health	59.6 ± 25.0	53.2 ± 26.1	<0.001*	0.25
Vitality	47.8 ± 22.4	63.0 ± 22.7	<0.001v	0.68
Social Functioning	69.2 ± 24.9	81.8 ± 22.6	<0.001*	0.53
Role Emotional	73.0 ± 27.4	76.3 ± 20.4	0.039*	0.14
Mental Health	60.4 ± 19.5	68.0 ± 18.3	<0.001*	0.40
PCS	53.2 ± 7.6	52.5 ± 6.9	0.142	0.10
MCS	43.6 ± 11.8	48.6 ± 9.2	<0.001*	0.48

SF-12v2 = The Short Form 12-Item Health Survey version 2; HK = Hong Kong; PCS = Physical Component Summary; MCS = Mental Component Summary;

Notes:

All parameters are expressed as mean ± sd.

Of 2,853 respondents in the study of population norm of the SF-12v2 in Hong Kong, 437 respondents could match our study subjects (on age and sex)

* Significant difference between doctors and HK general population (P < 0.05) by independent t-test or chi-square test as appropriate

a Cohen’s d effect size between doctors and HK general population

Compared to the HK general population, all domains of the SF-12v2 were statistically significant (P <0.05). Two domains were better in the doctor group: BP (84.5 (SD = 20.6) doctor group vs 79.9 (SD = 23.3) general population; P = 0.002; effect size 0.21) and GH (59.6 (SD = 25.0) doctor group vs 53.2 (SD = 26.1) general population; P = <0.001; effect size 0.25). The general population group scored higher in the remaining six domains. The largest effect sizes were noted for VT (0.68) and SF (0.53), with doctors scoring worse than the HK general population.

### Factors associated with HRQoL

The regression analyses are shown in **Table 3.** Better PCS scores were associated with regular exercise (Coeff = 2.024; 95% CI 0.026–4.023; P = 0.047). Worse PCS scores were associated with having children (Coeff = -1.890; 95% CI -3.656 –-0.124; P = 0.036) and higher personal burnout scores (Coeff = -0.045; 95% CI -.085 –-0.005, P = -0.027). In addition to depression being related to MCS (Coeff = -9.170; 95% CI -12.083 –-6.257; P<0.001), all three domains of the CBI burnout scale were related to MCS: personal (Coeff = -0.284; 95% CI -0.343– -0.224 P < 0.001), work-related (Coeff = -0.135; 95% CI -0.264 –-0.006; P = 0.040) and patient-related burnouts (Coeff = -0.060; 95% CI -0.117 –- 0.003; P = 0.041).

**Table 3:** Sociodemographic, professional satisfaction and health status associated with MCS and PCS by regression analysis.

Factor^a^	PCS (N = 309)			MCS (N = 309)		
	Coeff.	95% CI	P-value	Coeff.	95% CI	P-value
**Socio-demographic**						
Age	NA			NA		
Female (Vs Male)						
Married (Vs Single, separated and divorced)						
Having children (Vs No children)	-1.890*	(-3.656, -0.124)	0.036*			
Private setting of your Practice (Vs Public)	NA					
Current specialty						
Orthopedic Surgery/ Otorhinolaryngology/ Surgery/ Ophthalmology/ Obstetrics & Gynaecology						
Family Medicine/ General Practice/ Community Medicine, Emergency Medicine, Pediatrics						
Intensive care, clinical oncology, dermatology and venereology, internal medicine, psychiatry						
Pathology, radiology						
**Professional satisfaction**						
Not satisfied with your present job position (Vs Satisfied)						
Not satisfied with being a medical doctor (Vs Satisfied)						
**Health status**						
Average sleep per night						
Hours of work per week						
Current Smoker (VS non-smoker/ex-smoker)						
Regular exercise (Vs no regular exercise)	2.024*	(0.026, 4.023)	0.047*			
At-risk drinker	NA					
Depression (Vs No depression)				-9.170*	(-12.083, -6.257)	<0.001*
Burnout						
Personal	-0.045*	(-0.085, -0.005)	0.027*	-0.284*	(-0.343, -0.224)	<0.001*
Work-related	NA			-0.135*	(-0.264, -0.006)	0.040*
Patient-related				-0.060*	(-0.117, -0.003)	0.041*
Number of chronic illness				NA		

PCS = Physical Component Summary; MCS = Mental Component Summary; CI = Confidence Interval; Coeff = Coefficient

Notes:

Current Smoker (Current smoker vs Non-smoker/ex-smoker)

Regular exercise (5 or more days per week for at least 10 minutes per day / Any vigorous and moderate physical activities)

Private Practice (Private Solo/ Private Hospital/Non-government organisation

Public Practice (University/Government/Hospital Authority/Not applicable)

At-risk drinkers were defined if the doctors had 3 or more AUDIT-C scores. This AUDIT-C score was calculated by the sum of scores based on the frequency of drinking alcohol, the average number of standard drinks per day, and the frequency of taking 5 or more drinks on one occasion, ranging from 0 to 12. The frequency of drinking alcohol (Less than once per month/ Once per month/ 2 to 3 times per month or once per week/ 2 to 3 times per week/ 4 to 6 times per week or every day) ranks the score from 0 to 4 respectively; the average number of standard drinks per day (1–2 drinks/ 3–4 drinks/ 5–6 drinks/ 7–8 drinks/ 9 or more drinks) ranks the score from 0 to 4 respectively; the frequency of taking 5 or more drinks on one occasion (Never/ Less than once per week/ Monthly/ Weekly/ Daily or almost daily) ranks the score from 0 to 4 respectively.

* Significant with p-value < 0.05

a Variable in brackets is the reference category for independent variables

No data is shown for missing values.

As shown in **Table 4,** although males scored higher in all domains and summary scales than females, the differences were not statistically significant. There were significant differences in MH and MCS across age groups, specifically with MH (P = 0.042) and MCS (P = 0.012) between physicians aged less than 30 and physicians aged 35–39 years old (**Table 5**), but not in any of the other MCS or PCS domains.

**Table 4:** Differences in health-related quality of life between the different demographic groups.

		Physical Functioning		Role Physical		Bodily Pain		General Health		Vitality		Social Functioning		Role Emotional		Mental Health		PCS		MCS	
	N	Mean (SD)	P-value	Mean (SD)	P-value	Mean (SD)	P-value	Mean (SD)	P-value	Mean (SD)	P-value	Mean (SD)	P-value	Mean (SD)	P-value	Mean (SD)	P-value	Mean (SD)	P-value	Mean (SD)	P-value
Sex†	309	91.67 (18.39)	0.213	76.54 (26.11)	0.677	83.98 (20.70)	0.111	59.61 (24.55)	0.180	46.52 (21.84)	0.141	68.53 (25.21)	0.171	71.16 (27.75)	0.817	58.82 (19.97)	0.343	53.63 (7.36)	0.151	42.56 (12.18)	0.478
Male	174	92.82 (18.39)		77.08 (26.58)		85.63 (20.34)		61.26 (24.76)		48.13 (22.17)		70.26 (25.83)		71.48 (28.02)		59.77 (20.64)		54.16 (7.03)		42.99 (12.26)	
Female	135	90.19 (18.35)		75.83 (25.56)		81.85 (21.03)		57.48 (24.21)		44.44 (21.31)		66.30 (24.30)		70.74 (27.50)		57.59 (19.08)		52.94 (7.74)		42.00 (12.10)	
Age‡	309	91.67 (18.39)	0.232	76.54 (26.11)	0.831	83.98 (20.70)	0.728	59.61 (24.55)	0.068	46.52 (21.84)	0.094	68.53 (25.21)	0.066	71.16 (27.75)	0.066	58.82 (19.97)	0.042*	53.63 (7.36)	0.838	42.56 (12.18)	0.012*
<30	91	93.68 (15.63)		75.55 (26.93)		82.14 (22.45)		54.78 (25.64)		42.58 (21.89)		65.66 (25.99)		66.21 (29.26)		55.22 (19.10)		54.02 (6.79)		39.88 (12.03)	
30–34	95	92.37 (16.74)		75.39 (28.10)		83.95 (22.45)		60.37 (25.94)		45.79 (22.08)		67.63 (27.50)		70.00 (29.20)		58.16 (22.31)		53.83 (8.03)		41.97 (13.56)	
35–39	77	91.23 (18.03)		78.73 (24.51)		85.06 (18.25)		64.81 (22.29)		49.35 (20.67)		75.00 (21.07)		77.44 (24.34)		63.96 (17.56)		53.33 (7.51)		46.00 (9.46)	
>39	46	86.96 (25.68)		77.17 (23.17)		85.87 (17.20)		58.91 (21.70)		51.09 (22.33)		65.22 (23.85)		72.83 (25.59)		58.70 (19.16)		52.94 (6.91)		43.29 (12.45)	

PCS = Physical Component Summary; MCS = Mental Component Summary

Note

* Denotes statistically signiﬁcant results

† Using t-test

‡ Using ANOVA

**Table 5:** Differences in mental health domain and mental component summary by age post hoc comparisons.

Mental Health	<30	30–34	35–39
<30	-		
30–34	2.938	-	
35–39	8.741*	5.803	-
>39	3.476	0.538	-5.265
Mental Component Summary	<30	30–34	35–39
<30	-		
30–34	2.086	-	
35–39	6.120*	4.033	-
>39	3.406	1.320	-2.713

Mean differences are shown.

Note

* Denotes statistically signiﬁcant results

## Discussion

This study’s objective was to compare the HRQoL of HK physicians to the general population and to explore the factors associated with mental and physical HRQoL in HK physicians. The main findings from this study were that the doctors’ have poorer mental HRQoL compared to the general population, and HK doctors are more impacted by their mental problems than their physical problems. Regular exercise was associated with better physical HRQoL, but having children and higher burnout scores were associated with worse physical HRQoL. Poorer mental HRQoL was associated with higher burnout and depression scores.

Doctors in this study have similar PCS scores to the general population but statistically worse MCS scores, revealing that doctors’ mental health significantly impacts their lives. Compared to the only other HK physician study evaluating HRQoL, the mean MCS scores are similar, but the PCS score in this study is slightly better than theirs (53.2 (SD = 7.6) vs 49.6 (SD 7.6)) [31]. Since our data collection was done in 2016 compared to 2019 in theirs, this may reflect a decline in physicians’ physical health over time. Although their study was more recent, they did not evaluate the individualized domains of HRQoL nor did they assess the factors associated with HRQoL; whereas, we explored this in our study, and thus our results can be used to develop strategies to help improve doctors’ HRQoL.

We observed that the mean PCS was higher than MCS, implying HK physicians are less impacted by physical health problems than mental health problems. This is similar to the general HK population [41], where physical HRQoL is better than mental HRQoL and is also a trend seen in physician studies in HK, Mainland China, and Germany [29–31].

Overall, our study showed that HK physicians’ HRQoL is poorer than their Asian and global counterparts. Compared to a survey of Mainland Chinese doctors (Ding et al.) working in tertiary hospitals in the City of Shenyang, our study’s mean MCS and PCS scores were lower, implying HRQoL in HK physicians is worse compared to Mainland Chinese doctors [30]. A Norwegian (Stavem et al.) and a Singaporean (Tong et al.) physician study showed five and four of the eight domains were better in doctors than the general population, respectively [16, 61]. In contrast, in this study, only two domains (BP and GH) were better in physicians, although the effect sizes were small. Compared to both doctor studies, only the mean BP score was higher in our HK doctor group, and the remaining domain scores were lower in HK doctors. This may indicate that, overall, physicians’ HRQoL may be worse in HK compared to these countries.

However, comparisons with other studies may not be directly comparable due to some key differences. The Mainland Chinese study only looked at hospital-based doctors and did not include community doctors, compared to our current study, which included doctors in all settings and doctors not in clinical practice; thus, we may have greater generalizability. The Norwegian physician study examined a wider age range, sampled over 1000 doctors, and had two reference populations–University graduate and lower education level groups. Thus, they were able to show how education can have an effect on HRQoL and how age affects different domains. However, the effects of gender may be skewed because they had an overrepresentation of female physicians. The Singaporean study only studied doctors in a single hospital representing about 10% of the doctors on the island. However, they achieved a much higher response rate (83.2%) than us. Similar to our study, and thus making the analysis more comparable, their group of doctors was limited to a younger age group with a mean age of 33.6 years (SD 8.1).

Most of the domains under physical HRQoL were better in the doctors compared to the general population. Like participants in our study, BP and GH were higher in doctors than in age-matched participants with a lower level of education in the Norwegian physician study [16]. Studies have shown that higher education and socioeconomic status usually correlate with better health [62], which may explain the better GH domain in our study since physicians are well-educated. In addition, physicians’ work involves less physical labor than the general population, where 80% of those aged 25–39 years old in HK perform more manual work or have prolonged hours of standing [63]. Research supports that pain is less prevalent in highly qualified professionals with low degrees of manual labor than in more physically-taxing occupations [64] supporting our finding of lower BP scores in HK physicians than in the general population. PF had the highest mean score out of all the domains in this study, indicating that physical health does not limit the activities of HK physicians. It is likely because the age range of the participants was 24–45 years old in our study, indicating a younger and more physically healthy population as evidenced by their low levels of chronic illnesses. PF was also the highest mean score in the age-sex-matched general population of all the domains, supporting that a high PF may be related to age. In addition, this group of physicians had a high prevalence of regular exercise. This may mean they are more physically abled and less likely to have functional deficits related to their physical condition. Similarly, PF was also highest in younger physicians in the Singaporean study [61].

Although some physical health domains were better in physicians than in the general population, all mental health-related domains were significantly worse in physicians. In particular, VT, which had the lowest mean score, also had the highest effect size (0.68). This indicates that HK doctors have significantly more fatigue and less energy than the general population. Similarly, VT scored the lowest in physicians from four Southeastern European countries and Singapore [27, 61]. Reasons for this can be explained by physicians’ high prevalence of burnout [6, 49]. “Burnout is defined as the degree of physical and psychological fatigue and exhaustion perceived by the person”, which may be a similar measure to vitality [65]. During the development of the CBI, it was shown that there was a high correlation between personal burnout and VT [65]. In HK, 63.1–72.6% of physicians experience personal burnout [31, 43], explaining the low VT score. The strength of the study on Southeastern European countries was that there were over 1000 doctors sampled across five countries; however, they were limited to only general practitioners, which limits the generalizability of the data to other specialties.

SF score was lower in physicians than in the general population with a moderate effect size (0.53), meaning that HK physicians experience more limitations in their social life due to physical and emotional problems. Tensions between physicians’ personal and professional responsibilities, known as “work-home interference”, may directly affect these doctors’ social functioning [66]. Physicians in this study experienced longer work hours per week (55.2 hours) than the average HK worker (49.6 hours) [67]. Long hours mean less time to enjoy social activities contributing to diminished social functioning [43]. Research shows that doctors who are satisfied with their free time for non-medical activities have a better quality of life [35]. In the Singapore physician study, SF was also observed to be lower in physicians than in the general population. They suggested that it is a reflection that junior doctors have fewer social interactions and poorer social supports, which could be similar to young HK doctors [61]. MH score was lower in doctors in our study compared to the general population, with a low-moderate effect size (0.40) similar to the Singapore physician study. This could be related to higher burnout scores because research supports that higher personal and work-related burnout scores are correlated with lower MH scores [65]. Likewise, a significant proportion of the study population had positive screens for depression, which may affect the MH scores since it asks to rate the participants’ degree of feeling “downhearted and blue”, and low mood is one of the cardinal features of depression [50]. Lastly, RE was statistically worse in doctors than in the age-sex-adjusted HK general population. Even though the effect size was small (0.14), it seems to indicate that doctors’ emotional problems may affect their usual roles, such as those related to their work, but to a lesser extent than other mental health issues.

Concerning factors associated with HRQoL, depression and burnout were associated with lower MCS scores in HK physicians. Having children, having more burnout, and not having regular exercise were related to lower PCS scores. Studies show that MCS can be used to measure active and recent depressive disorder in adults in Europe [68] and HK [69], so it is no surprise that depression was associated with lower MCS scores in our study. The high prevalence of depression (15.2%) and its relationship to mental HRQoL is a problem worth addressing because we previously identified that physicians have poor help-seeking behaviors. Physicians have barriers preventing them from seeking treatment for their depression, which will also be a barrier to improving their physical and mental HRQoL [42]. In addition, all three types of burnout were related to lower MCS scores. Research shows that MH and VT, both low mental health domains in our study, strongly correlate with burnout [65]. SF, another mental health domain, was also found to be low in our study and may contribute to worsening burnout. Researchers have postulated that burnout affects workers’ family and social relationships and limits their ability to receive social support that may improve HRQoL [70]. It could also be possible that those with low perceived mental health show symptoms of burnout more or that certain environmental factors affect both burnout and HRQoL. Our findings that low MCS correlated with burnout were similar to a study of 168 medical residents working in a single hospital in Egypt. They found that higher burnout was associated with worse mental HRQoL [33]. However, unlike our study, the Egyptian study found that burnout was not correlated with physical HRQoL. Our current study found personal burnout was related to worse PCS scores [33]. The strength of the Egyptian study was that they were able to separate surgical from medical residents for comparison of HRQoL (where surgical residents scored lower scores in almost all SF-12 domains). Still, it only included residents from one hospital, which limits generalizability.

Our study found that doctors who performed regular exercise felt that their physical health did not limit their daily activities, as evidenced by higher PCS scores. However, exercise did not impact mental HRQoL. This was contrary to a study of Chinese medical students, which showed that more exercise was related to better results in mental and physical HRQoL [38]. However, studies on medical students may not correlate entirely to practicing physicians as stressors affecting mental health may differ. A systematic review of 14 studies found an association between exercise and HRQoL, with higher PF and VT being more consistently associated with higher exercise levels [71]. Some arguments proposed included a confounding construct, such as self-efficacy leading to a mediating step in the causal pathway between exercise and HRQoL, or that exercise and HRQoL may conceptually overlap, thus inflating the real association between these two constructs [71]. The discovery that MCS was not improved with regular physical activity in our study may limit the applicability of measures to use exercise to enhance HRQoL, but further research should be done to confirm the results.

Interestingly, having children was associated with worse PCS scores. There are no physician studies that we could find to compare to. However, because the physicians in this study were younger, it is assumed that their children were younger too. Parents of newborns, toddlers, and school-aged children tend to have poor sleep, which can manifest as poor physical health. Energy levels are typically lower with increasing sleep deprivation and feelings of physical exhaustion [72].

Analysis of physician gender and age and its effect on HRQoL showed that gender did not impact HRQoL. However, those aged 35–39 had better mental health scores than those less than 30 years old. This is similar to the Singapore study that showed senior doctors (over 30 years old) had slightly higher mental health scores than junior doctors (under 30 years old) [61]. Results from both the Singapore study and our study imply that younger doctors tend to display more symptoms of anxiety and depression and studies suggest that this may be due to long hours and less time relaxing during their early careers [61]. Similarly, the Norwegian physicians’ study showed that older physicians, particularly those 55–72 years of age, scored better in the mental health aspects of HRQoL. In addition, males significantly outperformed females in five of eight domains [16], whereas our study found that males outperformed females in all domains, but none were significant.

Last but not least, this study showed high professional satisfaction of HK doctors and did not find any relationship between PCS and MCS with professional satisfaction. This is in contrast to a Greek study that concluded nurses with high levels of job satisfaction had a better health-related quality of life [73]. Although nurses and the Greek healthcare system may not be similar to HK physicians, job demands may be similar. A study of young European surgeons [35] found that those who were satisfied with their work had a higher quality of life. Although the age range was not too dissimilar to ours, focusing on younger physicians (age 27–54 years old) and including specialist doctors and training doctors, this study used WHOQOL-BREF, which measures the quality of life and not HRQoL and only studied neurosurgeons, so it may not be directly comparable to our current study. The contrasting results may reflect slightly different effects of job satisfaction on HRQoL in HK.

Ultimately, doctors’ HRQoL may affect patient care. Research shows that clinical competencies are better for nurses who have fewer role limitations due to emotional problems, and have better mental health, social functioning, and physical functioning [74]. In addition, a Canadian study showed that for one-quarter of physicians, their physical or mental health problems made it difficult for them to handle their work in the short term; another one-quarter found that long-term physical, mental, or other health conditions reduced their work activity [13]. Therefore, it is concerning that HK physicians have fared poorly on many of the mental health dimensions compared to the general population and other doctors globally [16, 61] because it can affect their work.

To our knowledge, this is the first study that explored the factors affecting the HRQoL of HK doctors and compared the HRQoL of HK doctors to the general population. One key strength of our study is that we used an internationally validated measurement of HRQoL making it possible to compare our data to international studies. We also used age-sex-matched data to make direct comparisons with the general population. We also sampled doctors from different work settings (hospital vs community and private vs public health care), allowing for greater generalizability of our results. Our findings may help improve the understanding of physicians’ occupational health in a non-pandemic situation.

### Limitations

First, a causal association cannot be established with this cross-sectional survey. Second, there could be a response bias, as the original survey aimed to assess burnout and depression. Physicians with symptoms may be more or less likely to respond because the topic is relevant or because they are indifferent. Third, although we ascertained some of the study participants’ characteristics, we did not specifically ask for their years of experience. However, their age may grossly estimate their experience level. For statistical analysis, we also combined some of the related specialties that the participants’ were in as there were a small number of participants in some of the specialties. Fourth, despite two medical schools and foreign-trained doctors in HK, this study only sampled graduates from one medical school, limiting generalizability. However, for practical reasons, we only had the contact details of medical graduates from one school. Fifth, the participants’ ages ranged between 24–45 years old and may not reflect more senior doctors. Still, it was comparable to the age range of another HK physician study reporting on HRQoL [31]. Lastly, doctors in the private sector comprise about 48.9% of all doctors, but we only sampled 16.5% of private doctors [75]. This is because recent graduates in our setting undergo postgraduate training in the public sector. Data collection for this study was undertaken in 2016. With the COVID-19 pandemic, more up-to-date studies are needed to better understand how the pandemic has affected the HRQoL of doctors in HK.

## Conclusion

This study highlights that the HRQoL of HK physicians may be worse in HK compared to other countries. In addition, there may be a slight decline in physical HRQoL in recent years. Physicians in HK are more affected by their mental health problems than their physical health problems. Thus, it is essential to address depression and burnout in physicians, both highly prevalent concerns in HK and correlated with worse mental HRQoL in physicians. Strategies to improve HRQoL are necessary for the development and success of the healthcare system, as physicians’ HRQoL may affect patient care. Encouraging regular exercise and supporting doctors with children may help improve the impact of physical health problems on physicians.

## Abbreviations

AUDIT C: Alcohol use disorders identification test version C
BP: Bodily pain
CBI: Copenhagen Burnout Inventory
GH: General Health
HK: Hong Kong
HRQoL: Health-related quality of life
MCS: Mental component summary
MH: Mental health
PCS: Physical component summary
PHQ-9: Patient health questionnaire-9
PF: Physical functioning
RE: role emotional
RP: Role physical
SF: Social functioning
SF-12: Short Form 12-item Health Survey
SF-12 v2: Short Form 12-item Health Survey version 2
SF-36: 36-Item Short Form Survey
WHOQOL-BREF: World Health Organization Quality of Life: Brief Version
VT: Vitality
